# Learning stable radiation boundaries for wave simulations via passive neural state-space models

**DOI:** 10.1016/j.isci.2026.116116

**Published:** 2026-06-05

**Authors:** Aotu Li, Xiaolong Wang, Yuchen Wang, Shaoqiang Wang

**Affiliations:** 1 School of Science, Qingdao University of Technology, Qingdao 266520, China; 2 School of Information and Control Engineering, Qingdao University of Technology, Qingdao 266520, China

**Keywords:** physics, computer science, engineering

## Abstract

Accurate truncation of unbounded domains is a central challenge in time-domain electromagnetic (EM) systems. While perfectly matched layers (PMLs) can deliver excellent absorption, they typically require multiple grid layers and auxiliary variables, increasing runtime and memory costs. This work introduces a single-layer neural complete radiation boundary condition (NP-SSM-CRBC) that replaces a thick absorbing boundary with a one-cell boundary model. Instead of explicitly evolving auxiliary fields as in conventional PML or convolutional PMLs (CPMLs) formulations, the proposed approach employs a neural state-space model to predict the boundary ghost-cell responses directly. The method learns an effective, time-causal boundary operator and is designed to remain stable over long time horizons through explicit physical constraints. Experimental results demonstrate that the proposed method achieves low reflection levels with substantially reduced computational overhead. These results provide a foundation for extension to larger three-dimensional problems and more complex boundary geometries.

## Highlights

• A single-layer neural boundary replaces thick absorbing boundary regions
• Passive state space modeling ensures stable long horizon wave simulations
• Learns boundary operators through causal prediction of ghost cell responses
• Achieves low reflection with reduced memory use and faster computation

## Graphical abstract

**Figure:**
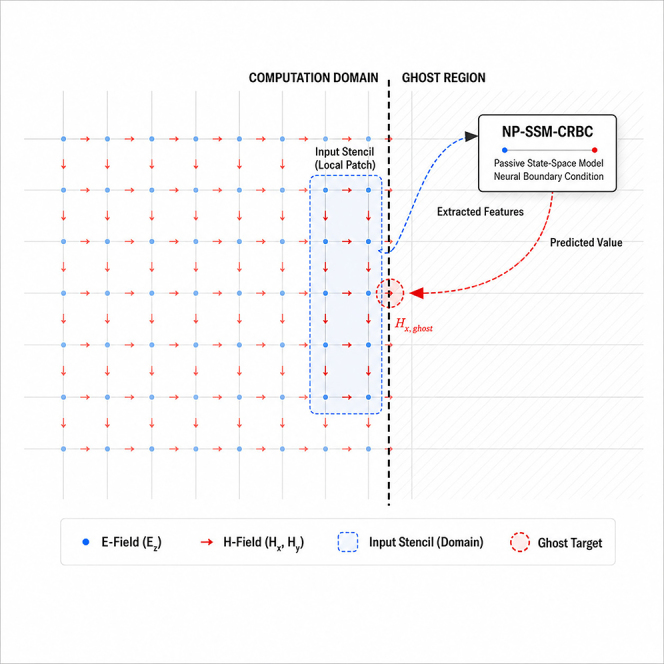

## Introduction

Numerical electromagnetics (EMs) based on finite-difference, finite-element, or finite-volume methods are cornerstones of modern scientific research and engineering design.^1^^,^^2^ A common challenge shared by these approaches is how to represent an inherently unbounded physical space within a finite computational domain.^3^^,^^4^ To address this challenge, artificial boundaries need to be equipped with absorbing boundary conditions (ABCs) or radiation boundary conditions (RBCs) that emulate the reflection-free outward propagation of EM waves.^5^^,^^6^

Early studies, such as the local non-reflecting boundary conditions,^7^^,^^8^ approximate absorbing operators by constructing high-order differential equations at the boundary. Although these methods are theoretically well grounded, their accuracy and angular robustness are often limited by the order of the underlying operators. Subsequent works utilized the concept of complete RBCs (CRBCs) and provided a more systematic theoretical framework for constructing high-accuracy and localized boundary conditions.^9^^,^^10^

Perfectly matched layers (PMLs) have been applied for truncating unbounded EM domains, due to their excellent absorption performance.^9^^,^^11^ By surrounding the computational domain with an artificial lossy medium exhibiting anisotropic material properties, PML can, in principle, achieve reflection-free absorption for incident waves of arbitrary angles and frequencies. However, this superior performance comes at a non-negligible cost. To attain sufficiently low reflection levels, PML typically require a thickness of 8–16 grid cells and carefully designed, gradually varying conductivity profiles.^12^^,^^13^ This significantly increases the total number of grid points in the simulation and necessitates the update of additional auxiliary differential equations (ADEs) or convolutional variables within the absorbing region, as in convolutional PMLs (CPMLs).^14^^,^^15^ As a result, both computational time and memory consumption increase substantially. Previous studies^16^^,^^17^ have also highlighted the practical complexity of configuring PML correctly, including the selection of layer thickness, grading profiles, and appropriate distances from evanescent or near-field sources.

A key motivation of this research is to focus on modeling capacity on the boundary closure operator itself, rather than replacing the entire unbounded exterior with a thick absorbing region. From a physical perspective, this operator corresponds to the Dirichlet-to-Neumann (DtN) mapping, which maps EM field values (Dirichlet data) on the boundary to their outward normal derivatives (Neumann data), thereby encoding the response of the entire exterior infinite domain. In the context of partial differential equations (PDEs) and stable control, boundary-to-boundary and boundary-to-flux neural operators have been shown to yield controllable and stable systems.^18^ In addition, the absorbing boundary operator in finite-difference time-domain (FDTD) methods can be interpreted as a time-domain convolution with long memory, which aligns naturally with the infinite impulse response structure of state-space models (SSMs).^3^ Beyond computational efficiency, SSM also provide stability properties, such as bounded-input bounded-output (BIBO) stability. These stability properties can be explicitly enforced through parameter optimization, while the key physical prior of passivity can be naturally incorporated.^19^

The main contributions are summarized as follows:

1. This work proposes a single-layer ABC based on a stable, parameterized SSM to approximate the infinite-domain DtN mapping. Instead of explicitly evolving auxiliary fields as required by conventional CPML formulations, the proposed approach employs a neural model to directly predict boundary ghost-cell responses, thereby realizing an effective boundary closure. An explicit passivity-regularized objective is introduced to constrain the boundary dynamics, preventing nonphysical energy injection into the computational domain and ensuring long-horizon numerical stability.
2. This work introduces an angle-adaptive low-rank tangential mixing mechanism to address boundary coupling induced by oblique wave incidence. By employing a lightweight mixing module with rank *K* = 8–16 along the boundary, absorption performance at large incidence angles is significantly improved with computational complexity *O*(*N* ⋅ *K*), avoiding expensive global operations such as the fast Fourier transform.
3. This work develops a unified boundary representation through coordinate transformations and symmetry exploitation, standardizing the four physical boundaries into a single right-boundary formulation. Within the proposed framework, a minimal 2 × 3 → 1/3 local stencil is employed to consistently handle edge points, endpoints, and corner points using shared parameters.
4. This work adopts a two-stage training and rollout stabilization strategy. In the first stage, the teacher forcing method with high-fidelity reference data is used to learn accurate local boundary responses. Subsequently, during the long-horizon autoregressive rollout fine-tuning stage, reflection-energy penalties and passivity regularization terms are introduced, effectively suppressing numerical drift caused by error accumulation.
5. This work demonstrates performance and cost advantages over CPML approaches. By replacing the explicit update of CPML auxiliary variables and absorbing regions of 10–20 grid layers with a single-layer boundary in which ghost-cell values are inferred by the neural model, comparable absorption performance is achieved while reducing memory consumption and providing runtime speedups of approximately 1.15× to 1.6 ×.

### Related work and background

Accurate treatment of artificial boundaries has long been a central topic in numerical EMs. Existing approaches can be broadly categorized into classical absorbing and radiation boundary conditions, virtual or operator-based absorbing boundaries, and learning-based boundary models. In parallel, recent advances in SSMs and stability-constrained neural architectures have provided new tools for addressing long-horizon dynamical systems. This section reviews related work along these directions and positions the present study within this evolving landscape.

#### Classical absorbing and radiation boundary conditions

Early developments in non-reflecting boundary conditions were established through the pioneering works in,^20^^,^^21^ which systematically derived hierarchical, convergent ABCs based on local differential operators. Higdon^22^ further investigated the construction of ABCs within finite-difference frameworks. Subsequently, the theory of CRBC, notably advanced by Hagstrom et al.,^9^ provided a rigorous theoretical and engineering foundation for constructing high-order boundary methods that systematically improve accuracy while preserving locality.

Since the introduction of PML by Bérenger et al.,^23^ PML-based techniques have become the dominant practical solution for domain truncation. The methodology has evolved from split-field PML to Unsplit PMLs (UPMLs), CPML, and high-order PML variants. Through careful material engineering and auxiliary variable recursion, these methods achieve extremely low reflection levels when sufficient layer thickness is employed. More recent developments, such as the RK-HO-PML method proposed in,^24^ further improve the efficiency and stability of high-order PML implementations and represent the current state-of-the-art among traditional absorbing boundary methods. Furthermore, Moura et al.^11^ introduced a formulation of complex frequency-shifted PMLs (CFS-PMLs) within the differential-forms framework, enabling efficient three-dimensional wave propagation scenarios.

#### Virtual absorbing boundaries and operator-based approaches

Beyond classical absorbing layers, alternative formulations have explored the concept of virtual or operator-based absorbing boundaries. A representative example is the virtual absorbing boundary (absorbing surface model, ASM) proposed in,^25^ which achieves near-zero boundary memory overhead by implementing absorbing behavior through operator-based formulations within a time-marching framework. In,^26^ Buraq et al. present an ABC defined by an operator tailored to absorb waves within a specified frequency band as an alternative to traditional PML for narrowband problems. Furthermore, Li et al.^27^ proposed a boundary-based Fourier neural operator surrogate that combines boundary element formulations with neural operators to enable efficient and accurate parametric wave analysis in exterior domains. Galkowski et al.^28^ construct local ABCs via Pade approximation of the DtN operator, enabling wave absorption directly at the boundary without introducing volumetric absorbing layers. This approach can be viewed as employing special absorbing elements confined to a single boundary layer, rather than relying on extended absorbing regions. The proposed method overcomes the scalability and conditioning limitations of conventional boundary element method and domain-based surrogate models. These works demonstrate that absorbing performance can be realized without explicitly extending the computational domain, highlighting the substantial engineering value and feasibility of the thin boundary paradigm.

#### Learning-based boundary conditions and stability-aware models

Learning-based boundary conditions have gained increasing attention in recent years. An early study^19^ demonstrated the feasibility of replacing multilayer PML regions with a shallow neural network operating directly at the boundary, achieving promising acceleration effects. Subsequent works,^29^^,^^30^ including long short-term memory-based PML variants,^29^ further explored the use of temporal sequence models to capture boundary dynamics. Compared with fully neural surrogates of entire simulators, boundary-only learning strategies are generally more lightweight, more stable, and easier to integrate with existing solvers.

In parallel, recent progress in control theory and operator learning introduced the concept of boundary learning, or boundary-to-boundary control. One study^31^ show that neural operators can learn mappings from boundary states to boundary fluxes, enabling safe and controllable boundary manipulation for PDE systems within finite domains. A representative work presented in one study^18^ provides both theoretical and experimental evidence supporting the feasibility of such boundary closure operators, offering independent validation of the underlying philosophy adopted in this study.

#### State-space models and stability-constrained neural dynamics

Recent advances in SSM, including Mamba^32^ and selective SSM architectures,^33^ have introduced a new class of linear-time sequence models that are both hardware-efficient and suitable for long-range temporal dependencies. The S5-PTD framework proposed in on study^34^ further established principled approaches to SSM initialization and parameterization, providing explicit guarantees on long-horizon stability. Such stability properties are particularly critical in physical simulations, where numerical drift and energy growth must be strictly controlled.

In the context of EM time-domain modeling, recent studies^35^^,^^36^ have demonstrated that causality-preserving neural operators based on temporal convolutions can achieve high accuracy while respecting physical constraints such as time causality and material dependence. These developments collectively suggest that stability-constrained state-space representations provide a natural and powerful foundation for modeling absorbing boundary operators in long-horizon EM simulations.

Existing approaches either rely on thick absorbing layers, lack explicit stability constraints, or treat boundary learning as a black-box surrogate, shown in Table 1. In contrast, the proposed approach unifies the advantages of operator-based boundary formulations and learning-based models within a single-layer framework. Unlike conventional PML and CPML methods that rely on thick absorbing regions and auxiliary variable updates, the proposed method directly learns a stable boundary closure operator by predicting ghost-cell responses, eliminating the need for volumetric absorbing layers. Furthermore, compared with prior learning-based boundary methods, explicit stability and passivity constraints are incorporated through a state-space formulation, enabling robust long-horizon time-domain simulations.

**Table 1:** Comparison of representative previous works and the proposed method

Method	Boundary type	Thickness	Operator	Learning-based	Stability Constraint
Engquist-Majda^20^	Local ABC	Thin	*✓*	×	×
Bayliss-Turkel^21^	Local RBC	Thin	*✓*	×	×
CRBC (Hagstrom)^9^	High-order RBC	Thin	*✓*	×	Partial
Pade-DtN ABC^28^	Local ABC (DtN-based)	Thin	*✓*	×	×
PML/CPML^23^	Absorbing layer	Thick (8–16 cells)	×	×	Implicit
RK-HO-PML^24^	High-order PML	Thick	×	×	Implicit
CFS-PML^11^	PML variant	Thick	×	×	Implicit
Virtual ABC^25^	Virtual boundary	Thin	*✓*	×	Partial
Operator ABC^26^	Operator ABC	Thin	*✓*	×	Partial
B-FNO^27^	Boundary surrogate	Thin	*✓*	*✓*	×
Neural PML^19^^,^^29^	Learned PML	Thick	×	*✓*	×
Boundary learning^18^^,^^31^	Boundary operator	Thin	*✓*	*✓*	Explicit
This work	Single-layer CRBC	One cell	*✓*	*✓*	Explicit (passive)

In highly heterogeneous media, PML performance could degrade due to impedance mismatch and numerical reflections at material interfaces, whereas the proposed method can adapt to complex media configurations through data-driven learning. From a scalability perspective, PML is mesh-independent and geometry-independent but requires additional volumetric absorbing layers, increasing computational cost. In contrast, the proposed NP-SSM-CRBC operates on a single boundary layer with a fixed local stencil, making it independent of domain size and more efficient for large-scale simulations. In terms of frequency response, PML is designed to provide near-uniform absorption across a broad frequency range, whereas the proposed data-driven approach offers the flexibility to adapt its absorption behavior through training. By incorporating diverse frequency components into the training data, the learned boundary operator can be tailored to specific frequency bands or complex wave scenarios, enabling more targeted and potentially more effective absorption in practical applications. Finally, PML may exhibit numerical instabilities in the presence of backward-propagating or evanescent waves under certain discretizations. By contrast, the proposed method enforces stability through a passive state-space formulation, providing a principled mechanism for stable time-domain boundary evolution. As a result, the proposed NP-SSM-CRBC achieves an effective balance between physical consistency, numerical stability, and computational efficiency.

#### Absorbing boundary conditions

In time-domain EM scenarios based on finite-difference, finite-element, or finite-volume methods, the computational areas need to be truncated to a finite region. To correctly represent outward-propagating EM waves without introducing nonphysical reflections at artificial boundaries, appropriate boundary treatments are required. Therefore, such techniques are commonly referred to as ABCs.^25^^,^^26^

From a mathematical perspective, an ideal ABC is equivalent to imposing an exact DtN mapping at the artificial boundary. The DtN operator corresponds to the complete response of the infinite exterior domain by mapping boundary field values (Dirichlet data) to their corresponding normal derivatives or fluxes (Neumann data). Because the DtN mapping fully encodes wave propagation in the unbounded exterior region, enforcing the exact DtN condition yields reflection-free boundary behavior. However, the DtN operator is generally nonlocal, time-dependent, and computationally expensive, which makes its direct implementation impractical for large-scale time-domain scenarios.

Classical local ABCs, such as the Engquist-Majda^20^ and Bayliss-Turkel^21^ formulations, approximate the DtN mapping by constructing local differential operators at the boundary. These methods achieve reasonable performance within limited frequency and incidence-angle ranges. More advanced approaches, including CRBCs,^9^^,^^10^ systematically improve approximation accuracy while maintaining boundary locality and a clearer theoretical connection to the underlying DtN operator. On the other hand, PML-based methods approximate DtN behaviors indirectly by surrounding the computational domain with artificial absorbing media and have become the dominant solution in engineering practice.

Despite their methodological differences, all of these approaches share a common objective: to approximate the effect of the infinite exterior domain through an effective boundary operator defined on a finite computational region. Within this context, the approach adopted in this work can be interpreted as a data-driven approximation of the DtN mapping. The proposed method provides a time-causal boundary closure operator that captures the dynamic feedback of the exterior domain on boundary nodes.

#### FDTD boundary updates and CPML mechanism

In FDTD method, EM field components are defined on a staggered Yee grid. For grid points located in the interior of the computational domain, field updates depend only on neighboring grid values. Near artificial boundaries, however, standard finite-difference stencils inevitably reference grid points that lie outside the computational domain. To preserve the consistency of update formulas, these virtual grid points are commonly referred to as ghost cells. Consequently, boundary treatment in FDTD can be equivalently viewed as the problem of providing a suitable numerical closure for ghost cells, such that the resulting updates accurately represent the physical response of the unbounded exterior domain.

For illustration, consider a one-dimensional transverse EM wave propagating along the *x*-direction. The standard FDTD update for the electric field component *E* at spatial index *i* and time step *n* can be written as:

(Equation 1) $$E_{i}^{n+1}=E_{i}^{n}+\frac{\Delta t}{\epsilon \;\Delta x}\left(H_{i+\frac{1}{2}}^{n}-H_{i-\frac{1}{2}}^{n}\right),$$

where Δ*t* denotes the time-step size, Δ*x* is the spatial grid spacing, *ϵ* represents the permittivity of the medium, and *H* is the magnetic field component defined at staggered spatial locations. When the index *i* is located adjacent to an artificial boundary, the update in Equation 1 depends on magnetic field values outside the computational domain, which must be supplied through appropriate ghost-cell values. This observation highlights that ABCs, at the discrete level, fundamentally correspond to specifying how ghost cells are computed.

However, in PML and CPML, ghost-cell behavior is not specified explicitly. Instead, additional auxiliary variables are introduced and updated recursively within the boundary region. In CPML formulations, for instance, the field update typically involves memory variables governed by recurrence relations of the form:

(Equation 2) $$\psi^{n+1}=a\;\psi^{n}+b\;\partial_{x}u^{n},$$

where *ψ* denotes a memory variable associated with the boundary, *u* represents the relevant field component, and *∂*_*x*_*u*^*n*^ is a discrete spatial derivative evaluated at time step *n*. The coefficients *a* and *b* are determined by the PML conductivity profile, the time-step size, and material parameters, and are chosen to enforce exponential decay of outgoing waves within the absorbing region. Equation 2 introduces a temporal convolution structure, effectively forming a linear time-invariant system with internal states that approximates the historical influence of the exterior domain.

From this perspective, CPML can be viewed not merely as an absorbing material model but as an explicitly designed boundary dynamical system that approximates the influence of the infinite exterior region. Its primary role is to provide a numerically consistent closure for ghost cells, ensuring that outgoing waves are absorbed rather than reflected. However, this mechanism relies on multiple auxiliary variables, empirically tuned parameters, and relatively thick absorbing layers, resulting in increased implementation complexity and computational overhead.

#### State-space models and stability

In time-domain EM scenarios, ABCs must not only suppress reflections at individual time steps but also maintain numerical stability over long time horizons. Since boundary updates are embedded into the global time-marching scheme through recursive relations, any unstable or nonphysical boundary dynamics may accumulate over time and ultimately lead to numerical divergence. Interpreting boundary updates from a system-theoretic perspective is therefore essential for constructing robust absorbing boundary formulations.

SSM provide a natural framework for describing discrete-time systems with internal memory. A general linear state-space representation can be written as

(Equation 3) $$x_{n+1}=A\;x_{n}+B\;u_{n},\;y_{n}=C\;x_{n}+D\;u_{n},$$

where x_*n*_ denotes the internal state of the system at time step *n*, u_*n*_ represents the input signal, and y_*n*_ is the output signal. The matrix A governs the temporal evolution of the internal state, B controls how inputs drive the state, C maps the internal state to the output, and D represents direct feedthrough from input to output. In the context of absorbing boundary modeling, u_*n*_ may correspond to tangential field components or their discrete derivatives evaluated at the boundary, while y_*n*_ is used to generate ghost-cell values or normal fluxes, thereby providing boundary closure for the interior update equations.

Within this formulation, the auxiliary-variable recursions employed in classical CPML schemes can be interpreted as manually designed linear state-space systems intended to approximate the historical response of the infinite exterior domain. However, if the spectral radius of the state transition matrix A is not properly controlled, or if the input-output mapping lacks physical constraints, the resulting recurrence would introduce numerical drift or instability during long-horizon simulations.

Stability of a discrete-time system is commonly characterized through BIBO stability. For linear systems, BIBO stability is ensured when the spectral radius of the state matrix A is strictly less than one, implying that any bounded input sequence produces a bounded output sequence. This condition provides an explicit and verifiable criterion for numerical stability of boundary recurrence models. In addition to stability, EM boundary models must satisfy passivity, meaning that the boundary should not inject nonphysical energy into the computational domain. Formally, a passive discrete-time system satisfies an energy inequality of the form:

(Equation 4) $$\overset{N}{\underset{n=0}{\sum }}u_{n}^{⊤}y_{n}\geq -E_{0},$$

where *E*_0_ denotes an upper bound on the initial stored energy of the system. This inequality guarantees that no net energy growth is introduced by the boundary during time evolution.

These concepts directly motivate the modeling choices adopted in this work. By representing the absorbing boundary as a parameterized SSM, the proposed NP-SSM-CRBC treats boundary closure as a dynamical system that approximates the exterior-domain response through learned ghost-cell evolution. Explicit constraints on the state-space parameterization are imposed to enforce BIBO stability and passivity.

## Results

### Proposed method

**Figure 1:**
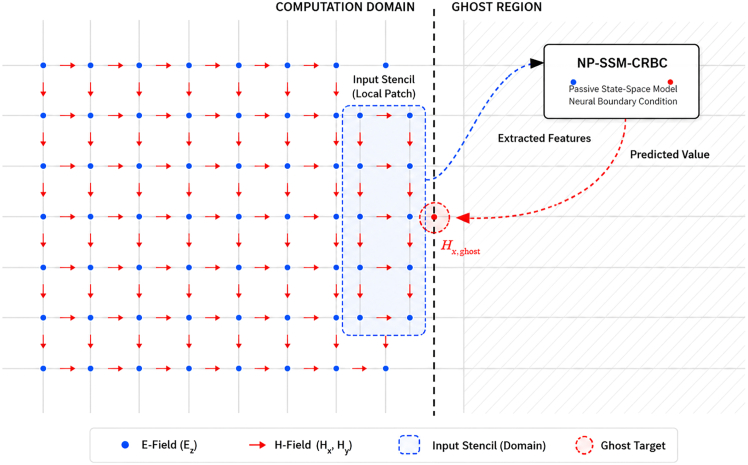
Schematic of ghost-cell prediction in the NP-SSM-CRBC framework

In FDTD scenarios, spatial updates near boundaries require field values outside the computational domain. These values are conventionally provided by auxiliary absorbing layers such as CPML, which introduce additional grid layers and memory variables. In this work, the boundary treatment is reformulated as a ghost-cell prediction problem: at each boundary grid point, the objective is to infer the field values in the immediately adjacent ghost cells, enabling standard FDTD updates without extending the computational domain. This ghost-cell prediction setup is schematically shown in Figure 1.

**Figure 2:**
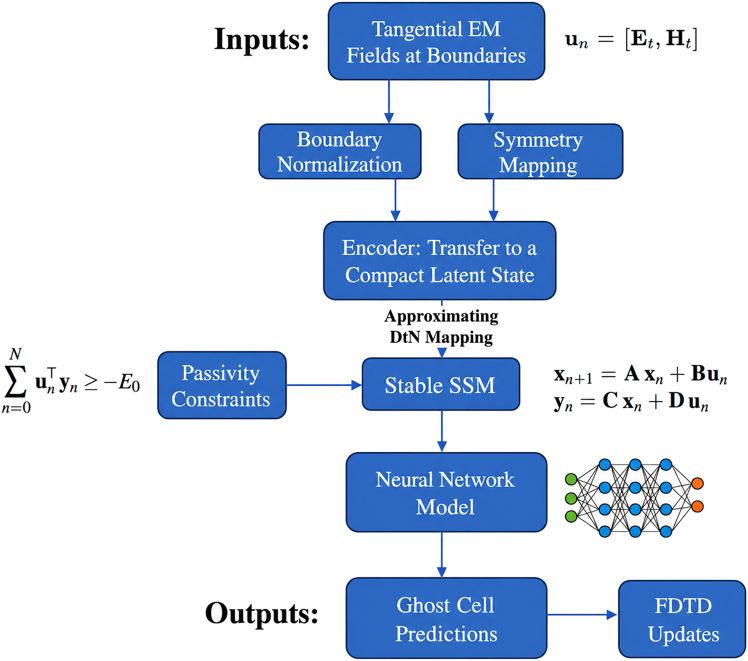
The proposed NP-SSM-CRBC framework

As shown in Figure 2, the tangential EM field components sampled at the artificial boundary are taken as input. These boundary fields are extracted from the finite-domain Yee grid at each time step. Firstly, they are transformed into a unified canonical representation through boundary normalization and symmetry mapping, allowing a single model to handle all physical boundaries consistently. A lightweight encoder then aggregates local spatial information into a compact latent representation suitable for temporal modeling.

The core of the method is an SSM model that approximates the DtN mapping of the infinite exterior domain. Figure 2 presents an overview of the proposed neural boundary modeling framework integrated with the FDTD scheme. Within the computational domain, local field values from a Yee-cell stencil adjacent to the artificial boundary are extracted to form the input features. These features are provided to a passive state-space neural model, which predicts the corresponding ghost-cell magnetic field values in the ghost region. The predicted ghost values are then fed back into the FDTD update equations, enabling stable field propagation while emulating an open-domain boundary condition without explicitly extending the computational domain. The SSM evolves an internal boundary state through a causal recurrence and produces boundary responses that reflect the accumulated influence of outgoing waves. To account for tangential coupling induced by oblique wave incidence, a low-rank mixing mechanism is incorporated along the boundary, improving absorption performance at large incidence angles while maintaining linear computational complexity. During training and rollout, explicit physical constraints described in Equation 4 are imposed on the boundary operator. In particular, passivity constraints are enforced to prevent nonphysical energy injection into the computational domain, thereby stabilizing long-horizon time-domain evolution. The final output of the proposed NP-SSM-CRBC framework consists of predicted ghost-cell field values, which are directly injected into the FDTD update equations, replacing conventional CPML boundary updates and eliminating the need for thick absorbing layers.

The predicted ghost-cell values are directly injected into the standard FDTD update equations. By replacing conventional CPML boundary updates with a single-layer, learned boundary operator, the proposed NP-SSM-CRBC framework achieves effective absorption with reduced computational and memory overhead while remaining fully compatible with existing FDTD solvers.

### Model architecture

#### Boundary canonicalization and stable causal design

To enable a single model to operate on all artificial boundaries of the computational domain (top, bottom, left, and right), a unified coordinate representation is introduced. Before being processed by the model, all local boundary sampling patches are transformed into an equivalent canonical right-boundary form through rotations, reflections, and component reordering. In this normalized coordinate system, the outward normal direction is consistently aligned, and tangential directions are arranged along the boundary. This unified representation significantly reduces the number of learnable parameters by allowing extensive parameter sharing across boundaries, and it ensures a consistent physical interpretation of boundary power flow. Under this normalized representation, tangential boundary fields are treated as a time series that drives causal state evolution.

For obliquely incident plane waves, boundary information propagates along the tangential direction, and purely local spatial stencils (e.g., 2 × 3) are insufficient to capture such long-range correlations. To address this limitation, a low-rank tangential frequency mixing mechanism is introduced along the boundary. This module enables information exchange between boundary points through separable one-dimensional convolutions or low-rank projections applied along the tangential direction.

Furthermore, boundary corner points connect two orthogonal boundaries and play a critical role in global numerical stability. To prevent abnormal energy accumulation at such locations, a power safeguard mechanism is introduced. At corner points, the model first predicts ghost-cell responses for the two orthogonal boundary directions separately and computes the corresponding outgoing power contributions. The predicted total outgoing power is then compared with the incoming power. If the outgoing power exceeds the physically admissible limit, the predicted responses in both directions are scaled proportionally to enforce the passivity condition. This safeguard mechanism provides robust local stability at corners without introducing additional state variables, thereby improving the overall robustness of the boundary model.

#### Prediction neural model

As shown in Algorithm 1, a lightweight prediction neural model is employed to map latent boundary representations to explicit ghost-cell field values. This module does not perform temporal modeling; instead, it serves as a feedforward mapper that converts causally accumulated boundary features into exterior field values required for FDTD updates. Specifically, the boundary input features are first normalized using a layer normalization without affine parameters to mitigate scale disparities among physical quantities. The normalized inputs are then processed by a MLP encoder composed of linear transformations and SiLU activations, projecting local boundary information into a unified feature space. The encoder output is subsequently fed into the SSM to drive causal state evolution.

Algorithm 1 **The proposed NP-SSM-CRBC model architecture**

**Input:** boundary input features $x\in R^{B\times d_{\text{in}}}$, previous SSM state $s\in R^{B\times d_{\text{state}}}$

**Output:** ghost predictions ${\hat{g}}^{\left(1\right)}$, ${\hat{g}}^{\left(3\right)}$, next state s^+^

//Input normalization

$\tilde{x}\leftarrow {LayerNorm}_{\text{no}-\text{affine}}\left(x\right)$;

//Neural encoder (multi-layer perceptron, MLP)

$z\leftarrow W_{2}\;\sigma \left(W_{1}\tilde{x}\right)\;$ with SiLU activation and dropout;

//Stable causal SSM update

Bz ←W_in_z;

*ρ* ← 0.999 *σ*(a), *θ* ← *π* tanh(w);

s^+^ ←ComplexRotate(s; *ρ*, *θ*) + Bz;

//SSM output projection with gating

c ←W_out_s^+^;

g ← *σ*(W_gate_z);

y_ssm_ ←c ⊙ g + W_skip_z;

//Residual fusion and normalization

h ←LayerNorm(z + y_ssm_);

//Ghost-cell prediction heads

${\hat{g}}^{\left(1\right)}\leftarrow W_{1}h$; //single ghost cell

${\hat{g}}^{\left(3\right)}\leftarrow W_{3}h$; //endpoint/corner ghosts

**return** ${\hat{g}}^{\left(1\right)},{\hat{g}}^{\left(3\right)},s^{+}$;

After the SSM update, a residual connection combines the encoded features with the SSM response, followed by layer normalization to stabilize the feature distribution. The prediction module adopts a multi-head output design to accommodate different boundary geometries. A single-output head is used for regular boundary points, producing one ghost-cell value, while a three-output head is employed for endpoints or corner locations, jointly predicting three neighboring ghost-cell values to provide richer local boundary information.

**Figure 3:**
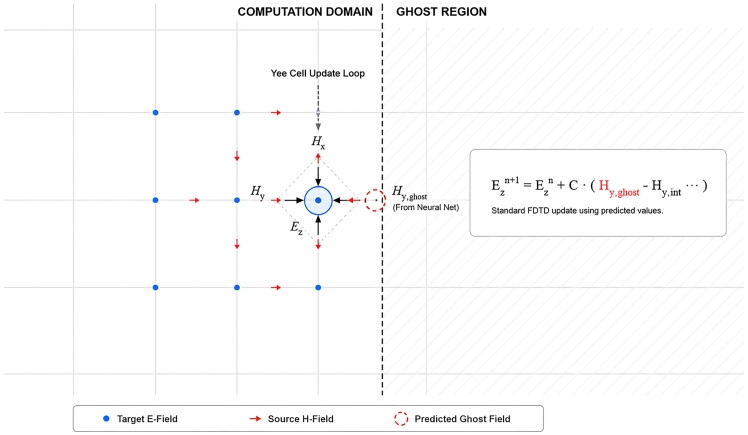
Interior field update using predicted ghost-cell values

Figure 3 illustrates how the predicted ghost-cell magnetic field values are incorporated into the standard FDTD update of interior electric fields near the artificial boundary. At each time step, the electric field component inside the computational domain is updated using neighboring magnetic field values defined on the Yee grid. For boundary-adjacent cells, one of these magnetic field components is replaced by the predicted ghost value provided by the neural boundary model. The resulting update preserves the standard FDTD time-marching structure while enabling the interior field to implicitly account for the influence of the truncated exterior domain. Its primary role is to transform physically constrained, time-aware boundary representations into ghost-cell values that can be directly injected into the FDTD solver, thereby replacing conventional CPML boundary updates.

### Dataset construction

**Figure 4:**
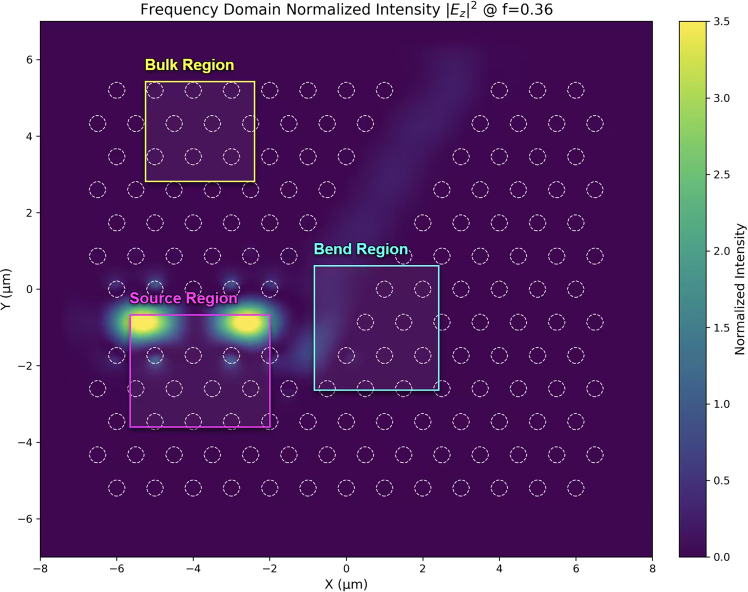
Photonic crystal waveguide benchmark used for region-diverse dataset construction

The dataset is constructed based on a representative two-dimensional photonic crystal waveguide benchmark with a 60° bend, as shown in Figure 4. This configuration is a classical FDTD test case that involves confined wave propagation along line defects in periodic media, accompanied by mode conversion and strong scattering at geometric discontinuities. Such characteristics make it a challenging and well-established benchmark for evaluating high-performance ABCs.

To enable a neural boundary model capable of replacing CPML under diverse physical conditions, a large-domain simulation followed by local domain cropping is adopted for dataset generation. Sampling is performed across three physically distinct regions highlighted in Figure 4. The source region (magenta) is located near the Gaussian pulse excitation and is characterized by high field amplitudes and steep spatial gradients, which trains the network to handle high-dynamic-range inputs and maintain numerical stability under strong excitations. The bend region (cyan) corresponds to the waveguide discontinuity, where severe scattering, directional changes, and higher-order modal components arise; samples from this region are critical for learning boundary responses to complex, nonplanar wave phenomena. The bulk region (yellow) is situated within the periodic lattice away from the waveguide and operates inside the photonic bandgap, where field amplitudes are weak and dominated by evanescent modes. These samples act as negative references, encouraging suppression of spurious reflections and preventing artificial field generation in background regions. By jointly sampling across these regions, the dataset captures a broad spectrum of physical regimes, enabling the neural boundary model to generalize effectively in complex photonic environments.

Specifically, the training dataset is constructed using high-fidelity time-domain EM simulations generated by the open-source FDTD solver Meep. To approximate an unbounded exterior domain with minimal reflection error, large computational domains equipped with thick PML are employed as reference simulations. In all experiments, PML regions with thickness *d*_pml_ = 12–16 grid cells and smoothly graded conductivity profiles are used to suppress boundary reflections and source interference.

Following Algorithm 2, each simulation episode produces a sequence of field snapshots

(Equation 5) $$U_{t}=Sim\left(\theta_{e};PML\right){|}_{t_{0}+t\Delta t},\;t_{0}=T_{\text{warm}},\;t=1,\ldots ,T,$$

where *θ*_*e*_ denotes randomized scene parameters, including material distributions, source locations, incidence angles, and excitation frequencies. After an initial warmup period of *T*_warm_ steps, field samples are collected with a temporal stride

(Equation 6) $$\Delta t∼Unif\left(\Delta t_{\min},\Delta t_{\max}\right),$$

which introduces temporal diversity while preserving causality.

Algorithm 2 **The dataset generation process using MEEP**

**Input:** $N_{\text{ep}},\;T,\;T_{\text{warm}},\;M,\;R=\left\{0,1,2,3\right\},\;d_{\text{pml}}$

**Output:** $D=\left\{X,Y,{mask}_{e},{mask}_{c},ep\text{\_}id\right\}$ saved as.npz

$S_{e}\leftarrow \emptyset ,\;S_{c}\leftarrow \emptyset$

**for** *e* = 1 **to** *N*_ep_ **do**

 Sample scene *θ*_*e*_ and run big-domain FDTD with PML(*d*_pml_) to get frames ${\left\{U_{t}\right\}}_{t=1}^{T}$ after warmup:

$$U_{t}=Sim\left(\theta_{e};\text{PML}\right){|}_{t_{0}+t\Delta t},\;t_{0}=T_{\text{warm}},\;\Delta t∼Unif\left(\Delta t_{\min},\Delta t_{\max}\right).$$

 Sample interior crop windows ${\left\{\Omega_{m}\right\}}_{m=1}^{M}$

 **for each** $\left(\Omega_{m},r\right)\in \left\{\Omega_{m}\right\}\times R$ **do**

$$\left({\tilde{U}}_{t}^{\left(r\right)},{\tilde{\Omega }}_{m}^{\left(r\right)}\right)=T_{r}\left(U_{t},\Omega_{m}\right),\;\left(X_{t}^{e},y_{t}^{e}\right)=\Phi_{e}\left({\tilde{U}}_{t}^{\left(r\right)},{\tilde{\Omega }}_{m}^{\left(r\right)}\right),\;\left(X_{t}^{c},y_{t}^{c}\right)=\Phi_{c}\left({\tilde{U}}_{t}^{\left(r\right)},{\tilde{\Omega }}_{m}^{\left(r\right)}\right),$$

 where $y_{t}^{c}=\left[g_{N},g_{E},g_{NE}\right]$ and sequences are stacked over *t* = 1:*T*

 $S_{e}\leftarrow S_{e}\cup \left\{\left(X^{e},Y^{e},e\right)\right\}$,

 
$S_{c}\leftarrow S_{c}\cup \left\{\left(X^{c},Y^{c},e\right)\right\}$

Let $N_{e}=|S_{e}|,\;N_{c}=|S_{c}|,\;N=N_{e}+N_{c}$

$$X=\left[\begin{array}{c} X_{e}\\ X_{c} \end{array}\right],\;Y\in R^{N\times T\times 3},\;Y_{1:N_{e},:,1}\leftarrow Y^{e},\;Y_{N_{e}+1:N,:,1:3}\leftarrow Y^{c},$$

$${mask}_{e}\left[i\right]=1\left(i\leq N_{e}\right),\;{mask}_{c}\left[i\right]=1\left(i>N_{e}\right).$$

Optionally: *X* ←clip(*X*, − *c*, *c*), *Y* ←clip(*Y*, − *c*, *c*)

Save np.savez_compressed(⋅) with $\left(X,Y,{mask}_{e},{mask}_{c},ep\text{\_}id\right)$

For each episode, multiple interior crop windows ${\left\{\Omega_{m}\right\}}_{m=1}^{M}$ are sampled from the large-domain simulation. Each crop is further transformed by a discrete rotation operator

(Equation 7) $$\left({\tilde{U}}_{t}^{\left(r\right)},{\tilde{\Omega }}_{m}^{\left(r\right)}\right)=T_{r}\left(U_{t},\Omega_{m}\right),\;r\in R=\left\{0,1,2,3\right\},$$

mapping all physical boundaries into a canonical right-boundary configuration. This rotation-based canonicalization enables a single boundary model to be trained across all boundary orientations.

At each time step, a local 2 × 3 spatial stencil immediately inside the artificial boundary is extracted as the input feature,

(Equation 8) $$x_{t}\in R^{2\times 3\times C},$$

where *C* denotes the number of physical channels, including tangential electric and magnetic field components and auxiliary material descriptors. The corresponding supervision signal is defined as the true ghost-cell field value located one grid layer outside the boundary,

(Equation 9) $$g_{t}\in R^{C},$$

obtained directly from the large-domain reference simulation.

For endpoint and corner locations, Algorithm 2 additionally extracts joint targets

(Equation 10) $$g_{t}^{\left(c\right)}={\left[g_{N},g_{E},g_{NE}\right]}_{t}\in R^{3\times C},$$

reflecting the coupled influence of orthogonal boundaries. Corner samples are intentionally oversampled to improve numerical stability near boundary intersections.

All samples are organized as temporal sequences of length *T*,

(Equation 11) $$D={\left\{\left(X_{i},Y_{i}\right)\right\}}_{i=1}^{N},\;X_{i}={\left\{x_{t}\right\}}_{t=1}^{T},\;Y_{i}={\left\{g_{t}\right\}}_{t=1}^{T},$$

where *N* is the total number of boundary samples aggregated across episodes, crops, and rotations. Boolean masks are stored to distinguish edge samples from corner samples during training. Optional clipping,

(Equation 12) X←clip(X,−c,c),Y←clip(Y,−c,c),

is applied to improve numerical robustness before saving the dataset in compressed.npz format.

In summary, the proposed data generation and training pipeline is designed to provide accurate and physically consistent supervision for learning a causal absorbing boundary operator. High-fidelity reference data are obtained from large-domain Meep simulations with thick PML layers, ensuring that the collected ghost-cell targets closely approximate the true exterior-domain response. Local boundary observations are extracted, normalized, and organized into temporal sequences to preserve the causal structure of wave propagation. The two-stage training strategy balances accuracy and long-horizon stability. Teacher forcing enables the model to learn precise local boundary responses, while the rollout fine-tuning stage exposes the model to closed-loop FDTD updates and suppresses error accumulation. Oversampling of corner and endpoint samples, together with extensive domain randomization, improves robustness under complex geometries and unseen excitation conditions.

All experiments must be conducted on Linux or WSL2 platforms using multi-threaded MEEP within a Conda-managed Python environment. The data generation code is publicly available and has been released alongside this work on the project’s GitHub repository to support reproducibility and further investigation.

### Experimental design

This section describes the experimental setup, training objectives, and efficiency analysis used to evaluate the proposed NP-SSM-CRBC framework.

#### Experimental setup

All experiments are conducted using two-dimensional time-domain EM simulations under transverse electric and transverse magnetic polarizations. High-fidelity reference solutions are generated with the open-source FDTD solver MEEP. A large computational domain is used to approximate an effectively unbounded space. Standard PML layers with thickness *L* = 12–16 grid cells are placed at the outer boundary to produce near-ideal teacher signals with minimal artificial reflections.

Excitations are Gaussian pulses with normalized amplitude, where the center frequency *f*_cen_ and bandwidth *df* vary across simulations. The incident angle is randomly sampled from 0° to 85°. The interior scene is randomized to include diverse material configurations, such as segmented media, waveguides, and scattering objects.

During each iteration, paired time series are recorded. The input is a local boundary stencil sampled inside the truncated domain using a 2 × 3 spatial window. The supervision target is the corresponding ghost-cell field located one grid layer outside the truncated domain. These synchronized sequences form the training dataset for learning a causal boundary operator. In the implementation, the spatial stencil is extended to a 2 × (2*r* + 1) patch with *r* = 2, corresponding to a 2 × 5 window that captures both normal and tangential field variations near the boundary. Additional features include finite-difference approximations of local derivatives and positional encodings along the boundary. Temporally, the model operates in a strictly causal manner, where the prediction at each time step depends only on the current local input and a compact recurrent state, without requiring a multi-step temporal window. This design ensures that the spatiotemporal input remains localized and computationally efficient.

#### Training strategy and optimization

A two-stage training strategy is adopted. In the teacher-forcing stage, the model prediction ${\hat{g}}_{t}$ is directly supervised by the reference ghost value *g*_*t*_ and optimized using the L2 regression loss.

(Equation 13) $$L_{\text{ghost}}=\|{\hat{g}}_{t}-g_{t}{\|}_{2}^{2},$$

which enables stable learning of accurate local boundary responses.

In the long-horizon rollout stage, the predicted ghost cells are recursively fed back into FDTD updates for multiple steps. The objective is extended with reflection-energy penalties and passivity regularization terms to mitigate error accumulation and suppress numerical drift. A scheduled sampling strategy is used to smoothly transition from fully supervised training to autoregressive rollout.

Optimization is performed using AdamW with an initial learning rate 3 × 10^−4^ and a cosine decay schedule. Mixed-precision training is enabled to accelerate training. Gradient clipping is applied with a threshold of 1.0. Training samples are organized as short time windows (e.g., 128 × 256) to balance temporal coverage and memory usage.

#### Loss functions and physics regularization

To explicitly suppress reflections at the boundary, characteristic variables are defined using Riemann invariants

(Equation 14) $$u^{\pm }=E_{t}^{\text{out}}\pm ZH_{z},$$

where *u*^+^ denotes the outward-propagating wave component, *u*^−^ denotes the inward (reflected) component, and *Z* is the local wave impedance. The reflection loss penalizes reflected energy:

(Equation 15) $$L_{\text{refl}}=E\left[|u^{-}{|}^{2}\right].$$

Passivity is enforced to prevent nonphysical inward energy injection. Based on the discrete Poynting theorem, inward power flux should be disallowed. The passivity loss is implemented as

(Equation 16) $$L_{\text{passive}}=E\left[\max\left(0,-E_{t}^{\text{out}}\cdot {\hat{H}}_{z}\right)\right],$$

where ${\hat{H}}_{z}$ is the predicted magnetic field component used in the boundary update. This term is consistent with the dissipative behavior expected from absorbing boundaries in FDTD.

Additional regularization terms can be included to encourage consistency with discrete Maxwell updates by substituting predicted ghost values into boundary curl/divergence relations and penalizing residuals. Stability is further supported by constraining the spectral radius of the SSM state matrix, typically used together with gradient clipping and weight decay.

#### Episode-aware training design

Algorithm 3 instantiates the practical training procedure for the boundary operator defined in Algorithm 1. An episode-aware training protocol is employed to ensure strict temporal separation between training, validation, and test data. All samples are grouped by simulation episode identifiers, and dataset splits are constructed by partitioning unique episodes rather than individual time steps. Input normalization statistics are computed exclusively from the training set and applied consistently across all splits. This design prevents temporal leakage and preserves the causal structure of wave propagation sequences. The NP-SSM-CRBC model is trained as a recurrent state-space system over a fixed horizon of length *T*. Given a normalized input sequence $\tilde{X}={\left\{{\tilde{x}}_{t}\right\}}_{t=1}^{T}$, the model evolves an internal state *s*_*t*_ and produces ghost-cell predictions at each time step,

(Equation 17) $$\left({\hat{y}}_{t}^{\left(1\right)},{\hat{y}}_{t}^{\left(3\right)},s_{t}\right)=f_{\theta }\left({\tilde{x}}_{t},s_{t-1}\right),$$

where ${\hat{y}}_{t}^{\left(1\right)}$ denotes the single ghost-cell prediction for regular boundary points and ${\hat{y}}_{t}^{\left(3\right)}$ denotes the joint ghost-cell prediction for endpoints and corner locations.

Algorithm 3 **Episode-aware training design of NP-SSM-CRBC**

**Input:** $D=\left\{\left(X_{i},Y_{i},m_{i}^{e},m_{i}^{c},{ep}_{i}\right)\right\}$, $X_{i}\in R^{T\times d}$; (*λ*_*e*_, *λ*_*c*_); *E*, *P*

**Output:** best EMA model and test metrics

**Split and norm:**

*E* ←unique(ep), *E* = *E*_*tr*_ ∪ *E*_*val*_ ∪ *E*_*test*_, *D*_*s*_ = {*i* : ep_*i*_ ∈ *E*_*s*_}

$\left(\mu ,\sigma \right)\leftarrow mean,std\left({\left\{X_{i}\right\}}_{i\in D_{tr}}\right),{\tilde{X}}_{i}=\left(X_{i}-\mu \right)/\left(\sigma +\epsilon \right)$

Initialize *θ*, EMA $\bar{\theta }\leftarrow \theta$, *best* ← + *∞*, *no*_*imp* ← 0

**for** *epoch* = 1 **to** *E* **do**

 **for each** $\left(\tilde{X},Y,m^{e},m^{c}\right)$ **do**

 *s*_0_ ← 0; **for** *t* = 1 **to** *T* **do**

 
$\left({\hat{y}}_{t}^{\left(1\right)},{\hat{y}}_{t}^{\left(3\right)},s_{t}\right)=f_{\theta }\left({\tilde{X}}_{t},s_{t-1}\right)$

$$L=\lambda_{e}\frac{1}{T}\underset{t}{\sum }\|{\hat{y}}_{t}^{\left(1\right)}-Y_{t}^{\left(1\right)}{\|}_{\text{SL}1}I\left(m^{e}\right)+\lambda_{c}\frac{1}{T}\underset{t}{\sum }\|{\hat{y}}_{t}^{\left(3\right)}-Y_{t}^{\left(3\right)}{\|}_{\text{SL}1}I\left(m^{c}\right)$$

 Update *θ*; $\bar{\theta }\leftarrow EMA\left(\bar{\theta },\theta \right)$

 Compute $L_{\mathrm{val}}\left(\bar{\theta }\right)$

 **if** $L_{\mathrm{val}}<best$ **then**

 $best\leftarrow L_{\mathrm{val}}$; save $\bar{\theta }$; *no*_*imp* ← 0

 
**else**

 *no*_*imp* ← *no*_*imp* + 1; **if** *no*_*imp* ≥ *P* **then**

 break

Evaluate test metrics using best $\bar{\theta }$

Supervision is applied conditionally using boolean masks to distinguish edge and corner samples. The edge and corner losses are defined as time-averaged SmoothL1 objectives,

(Equation 18) $$L_{e}=\frac{1}{T}\overset{T}{\underset{t=1}{\sum }}\|{\hat{y}}_{t}^{\left(1\right)}-y_{t}^{\left(1\right)}{\|}_{\text{SL}1}I\left(m^{e}\right),\;L_{c}=\frac{1}{T}\overset{T}{\underset{t=1}{\sum }}\|{\hat{y}}_{t}^{\left(3\right)}-y_{t}^{\left(3\right)}{\|}_{\text{SL}1}I\left(m^{c}\right),$$

and combined into a weighted objective

(Equation 19) $$L=\lambda_{e}L_{e}+\lambda_{c}L_{c}.$$

Optimization is performed using AdamW with cosine learning-rate decay, gradient clipping, and mixed-precision acceleration. An exponential moving average (EMA) of model parameters is maintained throughout training, and validation and test metrics are evaluated using EMA weights. The best model is selected based on the minimum validation loss with early stopping applied when no further improvement is observed.

#### Complexity analysis

A conventional PML with thickness *L* expands the updated grid from *N*_*x*_*N*_*y*_ to (*N*_*x*_ + 2*L*)(*N*_*y*_ + 2*L*). The number of additional updated cells introduced by PML is approximately.

(Equation 20) $$\left(N_{x}+2L\right)\left(N_{y}+2L\right)-N_{x}N_{y}.$$

For a 2D example with *N*_*x*_ = *N*_*y*_ = 512 and *L* = 12, the PML region contains 25, 152 additional cells. By contrast, the proposed NP-SSM–CRBC updates only a single boundary layer with approximately

(Equation 21) $$2\left(N_{x}+N_{y}\right)$$

cells. For *N*_*x*_ = *N*_*y*_ = 512, this corresponds to 2, 048 boundary cells, yielding a reduction of roughly 12.3× in boundary-update count.

Removing volumetric PML also reduces the total grid size from (*N* + 2*L*)^2^ to *N*^2^. The global update reduction ratio can be approximated as

(Equation 22) $$1-{\left(\frac{N}{N+2L}\right)}^{2}.$$

For *N* = 512 and *L* = 12, this yields ≈9.6% fewer global updates, and for *N* = 256 and *L* = 16, it yields ≈21.9% fewer global updates.

CPML methods also require updating multiple auxiliary memory variables per PML cell. In comparison, the proposed approach maintains a compact SSM state dimension *d* ≈ 8–16. The additional per-step cost is dominated by a low-rank tangential mixing module with complexity.

(Equation 23) O(N⋅K),K≤16,

where *N* is the boundary length.

Overall, the computational advantage of the proposed NP-SSM-CRBC method does not primarily stem from a reduction in floating-point operations. When measured purely in terms of arithmetic intensity, the neural state-space update introduces additional compute overhead compared to conventional CPML formulations shown in Equation 20. The observed performance gain instead arises from a fundamentally different memory access pattern. In conventional PML implementations, including CFS-PML and its time-domain realizations such as CPML, volumetric absorbing layers with depth *L* must be updated at each time step. This process involves repeated global memory access to multiple field components as well as auxiliary state variables associated with the boundary dynamics. In contrast, NP-SSM-CRBC operates exclusively on a single boundary layer and accesses only the local boundary fields together with a compact internal state S_*t*_. These quantities can be efficiently cached or retained in registers across time steps, significantly reducing global memory traffic. From a performance modeling perspective, the update of volumetric absorbing layers requires accessing multiple field components and auxiliary variables distributed across a relatively large memory footprint in state-of-the-art PML-based methods. This results in low arithmetic intensity and frequent global memory transactions, making the computation bandwidth-bound. In contrast, the proposed NP-SSM-CRBC operates on a compact boundary representation, where both the input stencil and the internal state S_*t*_ have small and fixed dimensionality. This allows most intermediate variables to remain in fast on-chip memory, thereby increasing effective arithmetic intensity and reducing memory access latency. As a result, the practical speedups observed in our experiments (approximately 1.15× to 1.6×) are primarily attributed to improved memory locality and reduced bandwidth pressure.

In classical absorbing layer approaches such as PML, the energy decay performance is dependent on the thickness *L* of the absorbing region, as energy dissipation is achieved through gradual spatial damping across multiple layers. Increasing *L* typically improves absorption by extending the propagation path within the lossy medium. In contrast, the boundary in the proposed NP-SSM-CRBCC framework is modeled as a single-layer operator that approximates the DtN mapping of the exterior domain. As a result, energy decay is decided by the dynamical properties of the learned SSM and the imposed passivity constraints, rather than by the length of the absorbing layer. From a computational complexity perspective, this distinction implies that the proposed method avoids the need for additional absorbing layers and their associated update costs, enabling efficient wave absorption with a constant-thickness boundary representation.

### Experimental results

This section presents a comprehensive experimental evaluation of the proposed NP-SSM-CRBC framework. Particular emphasis is placed on early-stage convergence behavior and its relationship to effective boundary absorption performance under a fully implemented training pipeline.

During the early stage of training, the validation reflection loss decreases rapidly and reaches

(Equation 24) $$L_{\text{refl}}^{\text{val}}\approx 6\times 10^{-4}$$

after the first training cycle. Based on the definition of the root mean square-equivalent reflection coefficient, this loss level corresponds to

(Equation 25) $$|\Gamma {|}_{\text{RMS}}\approx 2.3\%∼2.6\;\left(\text{approximately}\;-32\;dB\right),$$

indicating that the proposed NP-SSM-CRBC framework achieves effective boundary absorption already at an early stage of optimization. Compared with shallow learning-based ML-PML approaches in one particular study,^19^ which typically fail to surpass −20 dB in image-level relative error, the proposed method operates at a comparable or superior reflection level. This improvement primarily arises from the natural compatibility of the state-space formulation with time-domain wave propagation, as well as the stabilizing effect of explicit physical constraints.

The training, validation, and test loss curves further corroborate this observation. All three curves exhibit rapid decay within the first few epochs and remain closely aligned throughout training, with no visible divergence between data splits. After the validation loss saturates, early stopping is triggered, confirming that the optimization process is numerically stable and free from overfitting. This convergence behavior is consistent with the reflection-loss-based performance estimates and demonstrates that the learned boundary operator generalizes well under temporal rollout.

**Figure 5:**
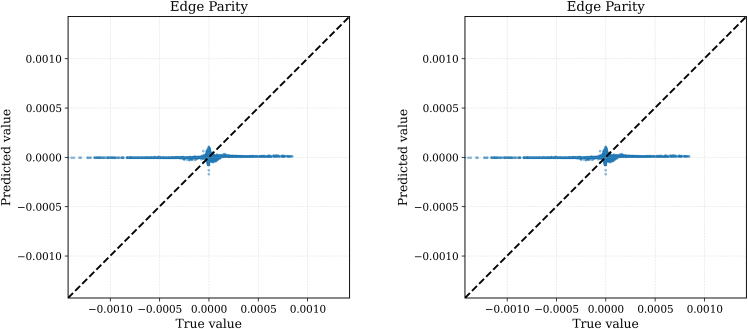
Ghost-cell prediction edge and corner parity plots

**Figure 6:**
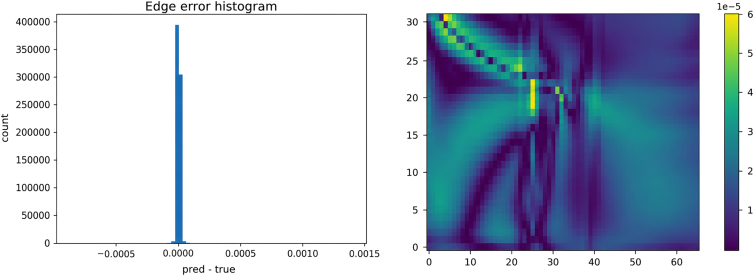
Ghost-cell prediction edge error histograms and heatmap

To assess boundary accuracy at the pointwise level, ghost-cell prediction parity plots, error distribution histograms, and the heatmap are analyzed, as shown in Figures 5 and 6. For edge locations, predicted values are tightly concentrated along the diagonal in the parity plot, indicating strong agreement with reference solutions. No systematic bias or directional asymmetry is observed, suggesting that the learned boundary operator does not introduce spurious energy injection or artificial reflections. The corresponding error histogram exhibits a sharply peaked distribution centered near zero, confirming that prediction errors remain small across a wide range of field magnitudes.

Corner locations require the joint prediction of three coupled field components and are therefore more challenging. However, the parity plots in Figure 5 and error histograms in Figure 6 indicate similarly stable behavior. The error distribution remains narrow and well-centered, with no evidence of heavy tails or numerical drift. The error heatmap shows that the absolute field error remains spatially localized near the boundary and corner regions without temporal amplification, indicating stable and physically consistent ghost-cell prediction. This observation is consistent with the low corner mean absolute error reported in the training logs and demonstrates that the proposed method maintains robustness even at geometrically sensitive boundary intersections.

### Ablation study and comparisons

To further examine the robustness and internal mechanisms of the proposed NP-SSM-CRBC framework, a series of ablation studies and comparative evaluations are conducted. These experiments are designed to isolate the contributions of key architectural components and training configurations and to assess model behavior under challenging propagation scenarios beyond the nominal training distribution. The ablation results are summarized in Table 2.

**Table 2:** Ablation study of the proposed NP-SSM-CRBC framework under different configurations

Configuration	State dim. *λ*	Stability constraint	|Γ|_RMS_ (dB)	Observation
Full model (default)	8	*✓*	≈−32	Stable, fast convergence
W/o stability constraint	8	×	≈−30	Occasional inward-growing fields
Increased capacity	16	*✓*	≈−33 to −34	+1–2 dB improvement
Large-angle incidence (>70°)	8	*✓*	Degraded (90th perc.)	Sensitivity at grazing angles
Strong scatterer near boundary	8	*✓*	Slightly degraded	Bounded but reduced stability

In long-horizon rollout tests, removing stability-oriented constraints from the state-space formulation leads to occasional “inward-growing” field patterns, which can trigger localized numerical instabilities under adverse conditions. Although such cases are infrequent, they highlight the importance of explicitly enforcing stability in the boundary operator. In particular, for large-angle incidence waves exceeding 70°, the reflection performance degrades noticeably, as indicated by an increase in the 90th percentile of the reflection coefficient |Γ|. This observation suggests that grazing-incidence scenarios impose stricter requirements on both model capacity and boundary representation.

To investigate the effect of model capacity, the state dimension *λ* is increased from 8 to 16. This modification results in an average improvement of approximately 1–2 dB in reflection performance, demonstrating a positive correlation between representational capacity and absorption effectiveness. Importantly, this improvement does not introduce additional numerical instability, indicating that the state-space formulation scales favorably with moderate increases in model complexity.

Additional stress tests are conducted in configurations where strong scatterers are placed in close proximity to the boundary. In these cases, intensified boundary-scatterer coupling partially reduces long-term stability and may lead to occasional energy accumulation. Nevertheless, the simulated fields remain bounded and no catastrophic divergence is observed. This behavior underscores the inherent robustness of the proposed approach while also delineating its operational limits under extreme coupling conditions.

From a comparative perspective, NP-SSM-CRBC achieves a favorable trade-off between absorption performance, numerical stability, and computational efficiency. Classical CPML and high-order PML schemes rely on carefully tuned absorbing layers of 8–16 grid cells to achieve extremely low reflection levels, at the cost of increased memory usage and auxiliary state updates. Frequency-domain approaches such as ASMs can approach theoretical absorption limits in large-scale spectral simulations, but are not directly compatible with explicit time-domain FDTD solvers. In contrast, NP-SSM–CRBC provides a fully time-domain, single-layer boundary formulation that integrates seamlessly into existing FDTD methods by replacing ghost-cell update rules.

## Discussion

This work introduces NP-SSM-CRBC, a neural complete radiation boundary condition that combines stable state-space modeling with explicit physical constraints to approximate the DtN operator of unbounded EM domains. By reformulating boundary absorption as a ghost-cell prediction problem, the proposed approach shifts modeling capacity from volumetric absorbing layers to a compact boundary-level operator. This design enables conventional multi-layer absorbing regions to be compressed into a single boundary layer, while preserving low reflection levels, numerical stability, and strict time causality. Extensive numerical experiments demonstrate rapid early convergence, accurate ghost-cell prediction at both edge and corner locations, and effective reflection suppression on the order of −32 dB in time-domain FDTD simulations. Beyond performance gains, an important implication of this work is the reinterpretation of ABCs as a learnable dynamical system with explicit stability and passivity constraints. This allows the learned operator to remain stable over long simulation horizons and mitigates error accumulation during autoregressive rollout, which is a critical requirement for time-marching solvers. From a systems perspective, the boundary operator learned in NP-SSM-CRBC can be viewed as a causal approximation of the exterior-domain response, providing a principled alternative to manually designed auxiliary-variable recursions used in CPML methods.

From a broader perspective, NP-SSM-CRBC achieves a favorable balance between traditional and modern strong baselines. Classical CPML and high-order PML methods rely on carefully tuned absorbing layers of 8–16 grid cells to achieve extremely low reflection levels, at the cost of increased memory consumption and auxiliary state updates. In contrast, the proposed method compresses the effective absorbing thickness from approximately 10-20 layers to a single boundary layer, as shown in Table 3, while attaining comparable early-stage reflection performance and significantly reducing the number of updated degrees of freedom. Compared with virtual absorbing boundary and operator-based frequency-domain methods, which approach theoretical absorption limits in large-scale frequency-domain solvers but are difficult to integrate into explicit time-marching schemes, NP-SSM-CRBC provides a fully time-domain-native solution. It can be seamlessly incorporated into existing FDTD solvers by replacing ghost-cell update rules without modifying interior field stencils. Furthermore, recent learning-based boundary control studies^18^^,^^30^ have demonstrated that learning boundary-to-boundary operators is an effective strategy for stabilizing PDE systems. NP-SSM-CRBC can be viewed as a concrete engineering realization of this philosophy within the Maxwell/FDTD framework, where learning capacity is concentrated on the high-leverage boundary operator and numerical stability is enforced through a causal state-space representation.

**Table 3:** Training log summary of NP-SSM-CRBC

Metric	Early stage	Best validation	Final stage
Epoch	10	45	45
Train loss	1.50 × 10^−1^	1.57 × 10^−5^	1.57 × 10^−5^
Validation loss (EMA)	3.03 × 10^−1^	1.48 × 10^−8^	1.48 × 10^−8^
Test loss (EMA)	3.25 × 10^−1^	4.28 × 10^−8^	4.28 × 10^−8^
Edge MAE (val)	2.99 × 10^−1^	1.01 × 10^−5^	1.01 × 10^−5^
Corner MAE (val)	6.06 × 10^−3^	6.25 × 10^−7^	6.25 × 10^−7^
Learning rate	1.0 × 10^−4^	5.8 × 10^−5^	5.8 × 10^−5^
Throughput (train)	∼3.0k/s	∼3.0k/s	∼3.0k/s

Moreover, the scalability of the proposed framework follows naturally from its boundary-local design. The learned operator acts on a compact spatiotemporal stencil defined at the boundary, which is independent of the global domain size. As a result, the trained model can be directly applied to larger computational domains without retraining, provided that the underlying discretization and physical scaling remain consistent. The training strategy further enhances robustness by incorporating diverse media configurations and excitation conditions, improving generalization across different scenarios. At the same time, the formulation assumes a fixed local stencil and a finite-difference discretization, where input features are constructed from local field values and their numerical derivatives. Consequently, significant changes in stencil size, boundary geometry, or numerical schemes would require adapting the input representation and retraining the model. This reflects a general trade-off in boundary operator learning between locality, generalization, and discretization consistency.

Another notable aspect of the proposed framework is its modularity and compatibility with existing numerical solvers. Because NP-SSM-CRBC operates exclusively at the boundary through ghost-cell prediction, it can be integrated into standard FDTD pipelines without modifying interior update stencils or time-stepping schemes. This property lowers the barrier to adoption and suggests that similar boundary-level learning strategies may be applicable to a broader class of wave-based simulations, including acoustics, elastodynamics, and other hyperbolic systems where artificial boundary truncation is required. Beyond EM simulations, it is worth noting that the proposed finite-difference methodology itself is widely used in other wave-based modeling domains, including acoustics and elastodynamics. From this perspective, the proposed passive state-space boundary formulation represents a more general methodological paradigm. Physically constrained dynamical models can be learned to replace handcrafted absorbing layers. Such a boundary-centric learning strategy may therefore offer a unified and extensible approach to artificial domain truncation across a broader class of hyperbolic systems. Another notable aspect of the proposed framework is its modularity and compatibility with existing numerical solvers. Because NP-SSM-CRBC operates exclusively at the boundary through ghost-cell prediction, it can be integrated into standard FDTD pipelines without modifying interior update stencils or time-stepping schemes. Beyond EM, it is worth noting that the proposed passive state-space boundary formulation can be viewed as a generalizable approach, where physically constrained dynamical models are learned to replace handcrafted absorbing layers. Therefore, the proposed boundary learning framework can be extended to other wave systems, including acoustic and elastic wave propagation problems.

### Limitations of the study

Despite these advantages, several limitations remain. The present study focuses on two-dimensional Maxwell equations with planar boundaries. Extending the framework to fully three-dimensional solvers introduces additional challenges, including increased boundary degrees of freedom, more complex tangential coupling, and higher computational cost. Curved or irregular boundary geometries may also require richer boundary representations or geometry-aware encodings to maintain absorption performance. Besides, the current approach relies on data generated from high-fidelity reference simulations equipped with thick PML layers. Although this strategy provides accurate supervision, it also inherits the assumptions and limitations of the reference boundary model. Incorporating structural priors from analytical CRBC formulations into the neural state-space parameterization is a promising direction to reduce data dependence and further improve interpretability. Future work will focus on extending the framework to three-dimensional Maxwell solvers and curved boundary geometries, where increased geometric complexity may require richer boundary representations. Incorporating structural priors from analytical CRBC formulations into the neural state-space parameterization is another promising direction to further enhance robustness and interpretability. In addition, hybrid approaches that combine the proposed time-domain boundary operator with frequency-domain or virtual absorbing boundary techniques may enable improved performance across broader frequency ranges and extreme incidence conditions. Furthermore, although the present study focuses on EM wave propagation, the proposed boundary operator learning framework is not inherently restricted to this setting. Extending the approach to elastic wave systems, where surface and guided waves such as Rayleigh and Lamb waves arise, introduces additional challenges due to more complex modal interactions and boundary effects. Addressing these scenarios would require dedicated training data and potentially enriched boundary representations. A systematic numerical investigation of Lamb wave absorption is therefore left for the future work.

## Resource availability

### Lead contact

Requests for further information and resources should be directed to and will be fulfilled by the lead contact, Yuchen Wang (wyc1990@qut.edu.cn).

### Materials availability

This study did not generate new materials.

### Data and code availability

• The datasets used for the experiments have been deposited at Mendeley Data: https://data.mendeley.com/datasets/khzkt7pn6p/1 and are publicly available as of the date of publication.
• The original code is available on GitHub: https://github.com/Blizzard-Passage-Lab/NP-SSM–CRBC.
• Any additional information required to reanalyze the data reported in this study is available from the lead contact upon request.

## Acknowledgments

Funding information is not available.

## Author contributions

Conceptualization, Y.W. and S.W.; methodology, A.L., X.W., Y.W., and S.W.; software, A.L. and X.W.; investigation, A.L. and X.W.; formal analysis, A.L. and X.W.; writing – original draft, A.L.; writing – review and editing, X.W., Y.W., and S.W.; funding acquisition, Y.W. and S.W.; resources, Y.W. and S.W.; supervision, Y.W. and S.W.

## Declaration of interests

The authors declare no competing interests.

## STAR★Methods

### Key resources table

**Table:** 

REAGENT or RESOURCE	SOURCE	IDENTIFIER
**Software and algorithms**		

Python (version ≥3.9)	Python Software Foundation	https://www.python.org/
NumPy (tested version = 1.24)	Python package	https://pypi.org/project/numpy/
SciPy (tested version = 1.10)	Python package	https://pypi.org/project/scipy/
PyTorch (tested version = 1.13)	Python package	https://pytorch.org/
torchvision	Python package	https://pypi.org/project/torchvision/
PyTorch Lightning	Python package	https://www.pytorchlightning.ai/
einops	Python package	https://pypi.org/project/einops/
PyYAML	Python package	https://pypi.org/project/PyYAML/
h5py	Python package	https://pypi.org/project/h5py/
tqdm	Python package	https://pypi.org/project/tqdm/
Matplotlib	Python package	https://pypi.org/project/matplotlib/
Seaborn	Python package	https://pypi.org/project/seaborn/
scikit-learn	Python package	https://pypi.org/project/scikit-learn/
Pandas	Python package	https://pypi.org/project/pandas/
Hydra-core	Python package	https://pypi.org/project/hydra-core/
OMEGACONF	Python package	https://pypi.org/project/omegaconf/
MEEP (FDTD solver)	MIT Photonic-Bands	https://meep.readthedocs.io/
MPI for Python (mpi4py)	Python package	https://pypi.org/project/mpi4py/
NP-SSM-CRBC codebase	GitHub	https://github.com/Blizzard-Passage-Lab/NP-SSM–CRBC

### Method details

#### Training settings

All experiments were conducted using Python and PyTorch. Model training was performed on a single NVIDIA GPU, with automatic mixed-precision enabled when supported. Random seeds were fixed for Python, NumPy, and PyTorch to ensure reproducibility. The model was optimized using the AdamW optimizer with learning rate LR and weight decay WEIGHT_DECAY. A cosine annealing learning-rate scheduler was applied over a maximum of MAX_EPOCHS epochs, with the minimum learning rate set to 10^−2^ times the initial value. The batch size was set to BATCH_SIZE. Gradient norms were clipped to CLIP_NORM to stabilize training. Early stopping was employed with a patience of EARLY_STOP_PATIENCE epochs based on validation loss. An exponential moving average of model parameters with decay factor 0.999 was maintained for evaluation.

#### Data generation and preprocessing

Training data were generated using two-dimensional finite-difference time-domain simulations with the MEEP solver. Large computational domains with perfectly matched layers were used to approximate unbounded electromagnetic space. For each simulation episode, randomized material geometries and excitation sources were generated inside an interior region of interest. After a warm-up period of T_WARMUP time steps, field snapshots were collected for T_STEPS steps at randomized sampling intervals. Multiple interior crop windows were extracted from each large-domain simulation. For each crop, boundary samples were collected along all four sides using rotational canonicalization. Tangential electric and magnetic field values from a two-layer interior stencil, together with discrete spatial derivatives and positional encodings, were used as input features. Corresponding ghost-cell values located one grid cell outside the cropped domain were extracted from the reference simulation and used as supervision targets. Corner samples were explicitly extracted and oversampled with a repetition factor of CORNER_REP. All input and target values were clipped to CLIP_VALUE. Input features were normalized using the mean and standard deviation computed from the training split only.

#### Model architecture

The NP-SSM-CRBC model consists of a boundary feature encoder, a neural state-space module, and output heads for edge and corner predictions. The encoder is implemented as a multilayer perceptron with N_LAYERS layers, hidden dimension D_MODEL, SiLU activations, and dropout. Temporal boundary dynamics are modeled using a neural state-space module with internal state dimension D_STATE and constrained spectral radius to ensure numerical stability. The model predicts one ghost-cell value for edge locations and three ghost-cell values for corner locations at each time step.

#### Training and evaluation

The model was unrolled autoregressively over the full temporal sequence length during training. Edge and corner predictions were supervised using a Smooth L1 loss, weighted by coefficients LAMBDA_EDGE and LAMBDA_CORNER, respectively. Training, validation, and test splits were constructed at the episode level to prevent temporal leakage. Model performance was evaluated using mean absolute error between predicted and reference ghost-cell values for both edge and corner locations. All reported results were obtained using the exponential moving average of model parameters.

### Quantification and statistical analysis

Quantitative evaluation of the proposed boundary model was conducted using the mean absolute error between predicted and reference ghost-cell field values for both edge and corner locations. Boundary absorption performance was quantified using reflection-related metrics derived from time-domain field data, including the root-mean-square equivalent reflection coefficient. Model robustness and numerical stability were assessed through long-horizon autoregressive rollouts, monitoring boundedness of field energy and the absence of numerical divergence. All metrics were computed on held-out test episodes using exponential moving average model parameters. Statistical analyses were performed in Python 3.9 using NumPy, SciPy, and PyTorch.
